# Reranking candidate gene models with cross-species comparison for improved gene prediction

**DOI:** 10.1186/1471-2105-9-433

**Published:** 2008-10-14

**Authors:** Qian Liu, Koby Crammer, Fernando CN Pereira, David S Roos

**Affiliations:** 1 Department of Computer and Information Science, University of Pennsylvania, Philadelphia, Pennsylvania, USA; 2 Google, Inc., Mountain View, California, USA; 3 Department of Biology, University of Pennsylvania, Philadelphia, Pennsylvania, USA

## Abstract

**Background:**

Most gene finders score candidate gene models with state-based methods, typically HMMs, by combining local properties (coding potential, splice donor and acceptor patterns, etc). Competing models with similar state-based scores may be distinguishable with additional information. In particular, functional and comparative genomics datasets may help to select among competing models of comparable probability by exploiting features likely to be associated with the correct gene models, such as conserved exon/intron structure or protein sequence features.

**Results:**

We have investigated the utility of a simple post-processing step for selecting among a set of alternative gene models, using global scoring rules to rerank competing models for more accurate prediction. For each gene locus, we first generate the *K *best candidate gene models using the gene finder Evigan, and then rerank these models using comparisons with putative orthologous genes from closely-related species. Candidate gene models with lower scores in the original gene finder may be selected if they exhibit strong similarity to probable orthologs in coding sequence, splice site location, or signal peptide occurrence. Experiments on *Drosophila melanogaster *demonstrate that reranking based on cross-species comparison outperforms the best gene models identified by Evigan alone, and also outperforms the comparative gene finders GeneWise and Augustus+.

**Conclusion:**

Reranking gene models with cross-species comparison improves gene prediction accuracy. This straightforward method can be readily adapted to incorporate additional lines of evidence, as it requires only a ranked source of candidate gene models.

## Background

Cross-species comparisons have been shown to be effective in locating genes and predicting gene structures. *De novo *gene finders such as SGP2 [1], TWINSCAN [2,3], NSCAN [4], SLAM [5], SAGA [6], DOGFISH [7], EXONIPHY [8], SHADOWER [9], CONTRAST [10] have improved upon *ab initio *gene finders through comparison with genomic sequences of reference species, capturing phylogenetic footprints, as coding sequences tend to be relatively highly conserved. Reference-based gene finders such as DPS [11], Rosetta [12], Procrustes [13], GenomeScan [14], Projector [15], GeneWise [16], GeneMapper [17] and ExonHunter [18] have sought to predict genes in target species through alignment with genes or proteins from reference species, modeling substitution patterns, gaps, exon/intron length distribution, signals, and other potentially conserved features. Augustus+ [19,20] extends Augustus [21] by incorporating alignments with genes and proteins of reference species into its *ab initio *gene model. FgenesH++ [22] also extends an existing *ab initio *prediction model with comparative evidence. Broadly speaking, all of these gene finders employ the strategy of adding comparative side-information to an existing *ab initio *model; genome annotation pipelines such as EnsEMBL [23] and UCSC Known Genes [24] add comparative components to *ab initio *models and expressed-sequence data sources. JIGSAW [25,26] employs a somewhat different strategy where *ab initio *and orthologous proteins are treated as sources of evidence and integrated. All of these gene finders effectively incorporate cross-species information, achieving improvement in prediction accuracy over single-species gene finders, although doing so often requires significant effort in model and algorithm design and implementation to cast comparative information into a form compatible with the existing gene models.

We have developed a simple, yet effective, reranking approach for incorporating cross-species information as a post-processing step after initial gene prediction, obviating the need to build a new gene finder or laboriously modifying an existing one to incorporate comparative information. Reranking the *K *best hypotheses has been an effective technique in natural language processing systems [27-29]. For example, in speech recognition, it is a widely adopted practice to generate the *K *best recognition hypotheses with a fast one-pass recognizer, and then rerank them based on probabilities given by a more powerful language model [30]. The gene finder Evigan [31] integrates diverse sources of evidence, yielding a ranked list of the top *K *candidate gene models, which may then be reranked by comparing them with reference genes from closely related species. Gene models with good (but not necessarily best) probabilities defined by Evigan that also exhibit strong similarity to reference genes may thus be selected as most likely.

## Results and discussion

To assess the feasibility and accuracy of reranking candidate gene models based on cross-species comparison, we conducted an experiment seeking to identify gene models in the genome of *Drosophila melanogaster *(stripped of all annotation), using *D. pseudoobscura *as reference species. *D. melanogaster *was selected because the extensive effort that has been devoted to gene annotation in this species provides a "gold standard" for assessing performance.

All data used in this experiment were downloaded from FlyBase [32], including:

• Whole genome sequence for *D. melanogaster *(Release 5.1), used as the target genome for gene model predictions.

• *Ab initio *gene model predictions from five gene finders (Augustus [21], Genscan [33], Genie [34], GeneID [35] and CONTRAST [10]), used as the input data for Evigan gene model predictions [31].

• Annotated gene models of *D. pseudoobscura *(Release 2.0), used as reference for reranking candidate gene models in *D. melanogaster*.

• Annotated gene models of *D. melanogaster*, used as training set for estimating reranker parameters, and also as a standard for evaluating prediction accuracy.

Evigan is a recently developed gene finder that integrates diverse sources of evidence, including predictions from multiple other gene finders. Using a dynamic Bayesian network to create consensus predictions based on the patterns of agreement and disagreement between the evidence sources, Evigan produces more accurate calls than any of the individual gene finders used as sources [31]. As output Evigan provides a list of the *K *gene models with the highest probabilities according to its evidence integration network.

Among the five source gene finders used as input for Evigan, four (Augustus [21], Genscan [33], Genie [34] and GeneID [35]) predict genes by examining the *D. melanogaster *genomic sequence and modeling the nucleotide composition surrounding start, stop, splice donor and splice acceptor sites, codon usage and coding potential, exon length distribution, and other sequence features. The CONTRAST [10] gene finder predicts *D. melanogaster *genes based on conservation with a reference species genome, motivated by the assumption that coding sequence is more likely to be conserved than non-coding sequence. Although CONTRAST uses genomic nucleotide sequence information from another species, none of the source gene finders uses gene models or proteins to improve gene model predictions.

**Figure 1:**
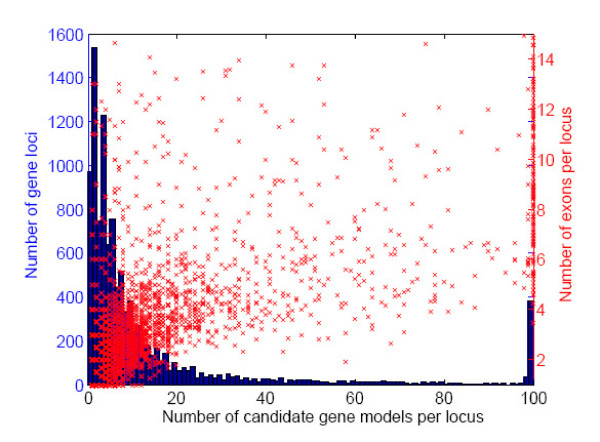
**Number of candidate gene models per gene locus and number of exons per gene on Drosophila melanogaster**. Blue bars provide a histogram showing the number of candidate gene models per locus, as identified by Evigan-5g. The red scatter plot shows the number of candidate gene models per locus versus the number of exons per gene (average number of exons per candidate where multiple candiates are predicted). Note that only a few candidate models are suggested for most genes; those with many candidate models predicted typically contain many exons.

Using the source gene finders' prediction sets, Evigan identified 13,669 gene loci in the *D. melanogaster *genome (see Methods). For each locus, Evigan was then used to generate the *K *best candidate models (*K *≤ 100), along with the probability for each model [31]. Figure 1 shows the number of candidate models identified per locus, and the number of exons per gene. Fewer than 20 candidate gene models were identified for 83% of the loci, although some loci contain as many as 100 competing models. In general, the number of plausible candidate models at a locus is a function of the number of exons for this gene: for loci exhibiting an average of < 5 exons per gene, Evigan identified a median number of 5 candidate models per locus; a median of 33 candidate models were identified for loci having an average of ≥ 5 exons per gene. The number of candidate models per locus identified by Evigan is based on the agreement among available evidence sources (gene finders). Disagreements about exon call multiply out for multi-exon genes, explaining the abundance of candidate models for those genes.

To identify genes where cross-species comparison might permit reranking of alternative gene models, *D. melanogaster *loci predicted by Evigan were filtered to identify those where: (*i*) Evigan suggested multiple candidate gene models, and (*ii*) putative orthologs (see "Methods") were identified in *D. pseudoobscura*. As indicated in Table 1, Evigan identified 13,669 genes in the entire *D. melanogaster *genome (some of the 14,550 genes curated in *D. malanogaster *release v5.1 were not recognized by any of the source gene finders, or only by a small subset, and were therefore not identified as probable genes by Evigan). Multiple candidate models were identified for 11,701 genes (86%), and 9125 genes (67%) were paired with *D. pseudoobscura *genes as putative orthologs based on reciprocal best BLAST hits [36]; 7975 loci exhibited both multiple candidate models and putative orthologs. A small sample (2.5%, 198 loci) of these genes were randomly selected as a training set for estimating reranking parameters, and the remainder (7777 loci) were used to test the reranking algorithm. Note that the five source gene predictors were trained on their specific training sets, but it is not very likely that they significantly overlap with the 198 loci randomly selected for estimating reranking parameters; otherwise the reranking parameters estimated from the training set would be biased and result in poor performance.

**Table 1:** Identification of D. melanogaster genes suitable for model reranking

*D. melanogaster *gene loci identified by Evigan-5g	13,669
Genes with multiple Evigan-5g candidate models	11,701

Genes with putative orthologs in *D. pseudoobscura*	9,125

Intersection (genes with multiple candidate models and putative orthologs)	7,975

Training set (2.5% of intersection, randomly selected)	1,98

Test set (used for Table 2)	7,777

Genes where ReRanker-5g selected the highest probability Evigan-5g model	6,031

Genes where ReRanker-5g selected a lower probability Evigan-5g model (used for Table 3)	1,746

The performance of Evigan-5g (which combines the five *ab initio *source gene finders) and of ReRanker-5g (which uses cross-species comparison to rerank the *K *best candidate gene models produced by Evigan-5g), were compared against curated annotation of the *D. melanogaster *genome (release 5.1). Performance metrics include sensitivity and specificity on the gene, transcript and exon level (see "Methods" for details), and the evaluation software Eval [37] was used. As indicated in the top section of Table 2, ReRanker-5g always performs better than Evigan-5g, in terms of both sensitivity and specificity, at the exon, transcript and gene levels for the genome, improving on the advantage that Evigan typically shows over any of the sources of evidence it integrates [31]. ReRanker-5g selected the highest probability Evigan-5g model for 6031 loci (by construction for these loci Evigan-5g and ReRanker-5g have the same prediction and thus the same performance); 4333 of these (71.9%), agree with the *D. melanogaster *genome annotation. Of the remaining 1746 loci where ReRanker-5g selected a lower probability Evigan-5g model, the highest probability Evigan-5g model was correct in only 252 cases (14.4%) whereas gene models selected by ReRanker-5g were correct for 500 cases (28.6%), indicating much better performance of ReRanker-5g than Evigan-5g. Results on these 1746 loci are shown in Table 3. The performance of Evigan-5g is relatively poor on these loci where genes contain relatively more exons (6.6 exons per gene on average for Table 3 versus 4.6 exons per gene for Table 2) reflecting the difficulties that genes of more exons pose to *ab initio *gene finders. Reranking candidate Evigan models based on sequence homology with *D. pseudoobscura*, however, significantly increases performance for these genes. When offered a selection of alternative gene models, cross-species comparison frequently allows ReRanker to select the correct models.

**Table 2:** Gene-finding performance for various algorithms.

	**Gene**		**Transcript**		**Exon**									
					**ALL**		*initial*		*internal*		*terminal*		*single*	

	sn%	sp%	sn%	sp%	sn%	sp%	sn%	sp%	sn%	sp%	sn%	sp%	sn%	sp%

Augustus	47.0	50.9	37.6	50.9	70.8	78.8	53.5	66.4	77.6	81.8	70.9	83.2	61.9	72.

CONTRAST	48.8	51.9	39.2	51.9	69.7	80.8	57.4	70.6	74.2	84.6	69.7	80.8	68.9	78.0

Geneid	35.9	41.4	29.3	41.4	65.7	71.4	47.0	60.9	75.6	73.9	59.2	72.8	54.6	73.7

Genie	40.7	50.0	31.9	50.0	58.2	77.9	44.1	63.7	63.1	82.7	58.8	80.2	58.7	68.8

Genscan	31.4	35.7	24.9	35.7	61.3	61.6	42.4	54.6	70.8	61.6	54.1	65.9	58.7	76.9

														

Evigan-5g	54.6	58.9	43.8	58.9	73.7	84.4	61.0	74.6	78.7	87.5	72.9	84.6	70.7	85.6

														

**ReRanker-5g**	**57.6**	**62.1**	**46.1**	**62.1**	**74.2**	**85.4**	**61.8**	**75.9**	**79.0**	**88.5**	**73.9**	**85.9**	**71.6**	**86.3**

														

GeneWise	29.4	31.0	25.0	31.0	58.3	73.9	41.8	56.7	69.5	*90.9*	48.5	59.4	32.3	30.6

Augustus+	53.3	57.0	43.5	57.0	73.0	81.1	58.3	72.2	*79.2*	84.0	71.6	83.3	65.2	73.0

Evigan-6g	56.3	60.7	45.1	60.7	*74.7*	85.2	61.4	75.4	*80.2*	88.3	73.5	85.7	70.5	84.7

Performance on the entire *D. melanogaster *test set of 7777 loci (see Table 1). Augustus, CONTRAST, Geneid, Genie and Genscan are *ab initio *predictors used as evidence sources for Evigan-5g. ReRanker-5g selects among K-best gene models produced by Evigan-5g with cross-species information. GeneWise, Augustus+ and Evigan-6g are other comparative gene predictors or approaches. Bold indicates where ReRanker-5g outperforms Evigan-5g; italics indicates where other comparative approaches outperform ReRanker-5g (see text).

**Table 3:** Gene-finding performance for genes where ReRanker-5g differs from Evigan-5g.

	**Gene**		**Transcript**		**Exon**									
					**ALL**		*initial*		*internal*		*terminal*		*single*	

	sn%	sp%	sn%	sp%	sn%	sp%	sn%	sp%	sn%	sp%	sn%	sp%	sn%	sp%

Evigan-5g	11.9	14.4	8.7	14.4	64.5	76.8	42.3	58.4	73.3	81.5	55.8	73.1	3.6	17.4

														

**ReRanker-5g**	**23.6**	**28.6**	**16.6**	**28.6**	**66.11**	**79.6**	**45.1**	**63.0**	**73.9**	**83.5**	**59.3**	**77.9**	**13.5**	**64.3**

														

GeneWise	18.8	21.5	14.4	21.5	56.4	75.7	32.5	49.2	66.9	*88.5*	41.2	55.9	9.9	14.7

Augustus+	*30.2*	*33.5*	*21.9*	*33.5*	*67.4*	76.2	*46.7*	62.2	*75.3*	80.1	*59.8*	74.3	*16.3*	23.0

Evigan-6g	19.0	23.1	13.7	23.1	*67.5*	79.1	43.8	60.7	*76.8*	83.3	58.7	77.3	2.8	17.4

Performance on the 1746 loci where ReRanker-5g selected a lower probability Evigan-5g model based on cross-species comparison. Note that ReRanker-5g improves on Evigan-5g acrosss the board; italics indicates where other comparative approaches outperform ReRanker-5g (see text).

**Figure 2:**
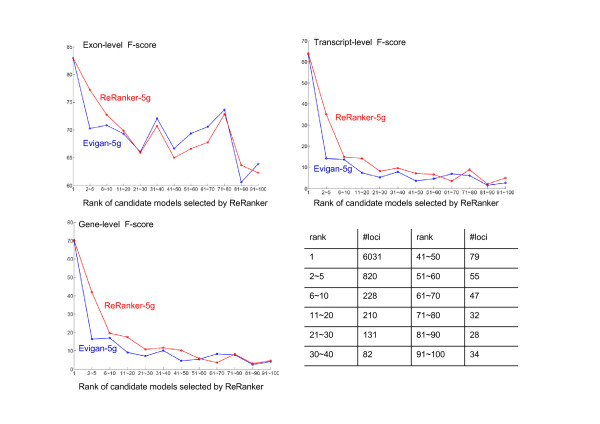
**Performance by rank on Drosophila melanogaster**. The table on the bottom right shows the number of loci where ReRanker-5g selects Evigan-5g candidate gene models of certain rank. For example, there are 6031 loci where ReRanker selects the most probable candidate models as defined by Evigan; there are 820 loci where ReRanker-5g selects the second to the fifth most probable candidate models as defined by Evigan, and so on. The other panels show the F-score (harmonic mean of sensitivity and specificity) of Evigan-5g and ReRanker-5g at the exon, transcript and gene levels for various rank ranges. ReRanker is successful at improving the identification of correct gene models even when selected candidates are far from the top of the list provided by Evigan.

When ReRanker selects an alternative model, does it always choose the next most probable candidate from the list of possibilities defined by Evigan? Figure 2 presents the frequency and performance of ReRanker selections, as a function of Evigan rank. ReRanker selected the second to the fifth most probable Evigan model in 820 genes, the sixth to the tenth most probable model in 228 genes; and even lower probability models for 698 genes. Comparison with the annotated *D. melanogaster *genome indicates that even when relatively low ranking models were selected by the reranking algorithm, these are more likely to be correct than the top probability Evigan model: the red lines (ReRanker-5g) are higher than blue lines (Evigan-5g) in Figure 2 for all exon, transcript and gene levels.

Incorporating Genie's prediction into ReRanker-5g (through Evigan-5g) could have introduced a circularity, because ReRanker's performance was evaluated on *D. melanogaster *annotations, which were developed with the help of Genie. However, this does not appear to be the case, since both ReRanker-5g and Evigan-5g significantly outperform Genie for sensitivity and specificity on the gene, transcript and exon level, as shown in Table 2. In fact, ReRanker-5g and Evigan-5g significantly outperform all of the five *ab initio *predictors used as evidence sources for Evigan-5g (Table 2).

Another factor that migh raise concerns of circularity in our evaluations is ReRanker's use of *D. pseudoobscura *as a reference for gene prediction on *D. melanogaster*, since the former was annotated based on the latter. To address this, performance of ReRanker-5g and Evigan-5g were evaluated on 1191 *D. melanogaster *loci whose putative orthologs on *D. pseudoobscura *have EST support. Over 38,000 EST sequences for *D. pseudoobscura *were obtained from dbEST [38] and aligned to *D. pseudoobscura *annotated transcripts that were identified as the putative orthologs of the entire test set (7777 transcripts), using BLAST (E-value cutoff of 1e-5). The transcripts where the aligned length covers more than half of the transcript length were retained as a subset having independent experimental support, resulting in 1191 transcripts. On the *D. melanogaster *loci which are these transcripts' putative orthologs, performance of ReRanker-5g and Evigan-5g were evaluated and presented in Table 4. ReRanker-5g outperforms Evigan-5g for sensitivity and specificity at the gene, transcript and exon level. The evaluation on the subset with independent experimental evidence suggests that ReRanker's improved performance is not likely to be attributed to the two species' related annotation process.

**Table 4:** Gene-finding performance for D. melanogaster genes with D. pseudoobscura EST evidence.

	**Gene**		**Transcript**		**Exon**									
					**ALL**		*initial*		*internal*		*terminal*		*single*	

	sn%	sp%	sn%	sp%	sn%	sp%	sn%	sp%	sn%	sp%	sn%	sp%	sn%	sp%

Evigan-5g	71.0	74.6	59.2	74.64	78.1	88.9	72.0	86.3	81.8	90.0	77.9	90.0	81.4	89.2

**ReRanker-5g**	**75.6**	**79.4**	**63.2**	**79.4**	**79.4**	**91.2**	**74.0**	**88.8**	**82.5**	**92.7**	**79.8**	**91.8**	**81.8**	**89.6**

Performance on the 1191 *D. melanogaster *loci whose putative orthologs on *D. pseudoobscura *are supported by EST sequences (see text for details). Note that ReRanker-5g improves on Evigan-5g across the board (improvement indicated by bold).

A widely used alternative approach for using cross-species information in gene prediction involves aligning reference gene models (or proteins) to the target genome, and using these alignments to either build new gene finders, or modify existing *ab initio *ones by explicitly modeling alignments. GeneWise [16] aligns protein sequences to target genome sequences and uses the alignments to hypothesize introns, amino acid mutation patterns, sequencing errors, exon length statistics, and other gene prediction signals.

Augustus+ [20] extends the *ab initio *gene finder Augustus [21] by considering transcript or protein alignments as extrinsic hints, up- or down-weighting *ab initio *gene parses based on consistency with the alignments. The bottom half of Tables 2 and Table 3 compares the performance of ReRanker-5g with GeneWise and Augustus+ on the complete *D. melanogaster *test set, and the subset of genes where Evigan-5g and ReRanker-5g chose different models. GeneWise was employed to align *D. pseudoobscura *proteins to their putative orthologous loci in *D. melanogaster *(using default parameters). GeneWise predictions of CDS, donor, acceptor, start and stop information were then provided as extrinsic hints for Augustus+ (using *ab initio *parameters trained for *Drosophila melanogaster *and default parameters for extrinsic protein hints). Evigan was also run to integrate GeneWise models with the five *ab initio *source gene finders described above, yielding Evigan-6g.

Augustus+, GeneWise and some other comparative predictors do not need ortholog detection; rather they align reference genes or proteins to a target genome and then refine signal predictions for significant hits. This strategy tends to identify relatively more target genes and thus enjoy higher sensitivity. For ReRanker, where putative ortholog detection is needed, if ortholog detection for a gene fails, ReRanker misses the opportunity to locate the gene and will thus show lower sensitivity. However, ReRanker's main goal is to improve specificity by improving the prediction of exact structures for genes whose existence and rough locations have been reasonably validated.

On the whole *D. melanogaster *test set (Table 2), ReRanker-5g outperformed GeneWise and Augustus+ in terms of both sensitivity and specificity, at the exon-, transcript-, and gene-level (although GeneWise exhibited slightly greater specificity, and Augustus+ slightly greater sensitivity, in recognizing internal exons). ReRanker-5g also outperformed Evigan-6g as assessed by all criteria except for overall and internal exon sensitivity. In cases where ReRanker-5g and Evigan-5g make different choices (Table 3), ReRanker-5g outperforms GeneWise and Evigan-6g, but performs worse than Augustus+ in most categories. The better performance of Augustus+ (on this subset of genes, but not the genome as a whole; Table 2) may arise from increased sensitivity by using homology information in its *ab initio *model used to search for gene segments. The relatively poor performance of ReRanker-5g would then follow from a relative lack of candidates in the source evidence: ReRanker-5g is constrained to select from among the potential models suggested by Evigan-5g, which performs relatively poorly on this subset. These observations highlight the extent to which Evigan and ReRanker are limited by available sources of evidence, in particular the source gene finders, but it is important to note that the inclusion of additional gene predictions in the mix is likely to improve the performance of Evigan and thus ReRanker.

## Conclusion

We have demonstrated that ReRanker leads to improvement in prediction accuracy through a simple strategy of incorporating additional evidence. There are many directions along which the work can be extended or improved. The first step of the reranking strategy is to identify single-gene loci on a target species. If this step finds incorrect loci, such as loci that contain more than one gene, partial genes or pseudo genes, it could mislead ReRanker, which assumes that a locus contains a single gene. In the ortholog identification step, most of those wrong loci will be removed because they tend to not be associated with orthologs from a reference species. But it would be very useful to devise an additional step before reranking, to identify problematic loci and even recover correct locus information. The reranking strategy is sufficiently general, in the sense that it is neither specific to Evigan candidate gene models, nor limited to incorporating information from cross-species comparisons. The same conceptual strategy could readily be applied to candidate gene models produced by other annotation pipelines as well as accommodate diverse sources of evidence in place of or in addition to comparative genomics data. For example, one can easily envision further improving gene models selection by reranking based on protein sequence motifs or signals, transcript or protein expression data, etc. In addition it is natural to relate Evigan's K-best gene models to alternative transcripts, which might allow us to extend ReRanker for predicting multiple transcripts on a target species, if the putative ortholog on a reference species exhibits alternative transcripts.

## Methods

This section details how ReRanker prioritizes candidate gene models on a target species by comparison with orthologs from a reference species. Subsections address the generation of candidate gene models, ortholog identification between the two species, the construction of similarity features between gene models, the format of scoring function of candidate gene models and learning of the reranker's scoring parameters.

### Generating candidate gene models

Gene loci on the target species were first defined and candidate gene models for each locus are generated by Evigan. The term gene locus refers to a genomic region containing only a single gene. Gene loci on the target species were first identified by an initial prediction gene set produced by Evigan integrating multiple lines of evidence (Augustus, Genscan, Genie, Geneid, CONTRAST were used in the experiment). The genomic region defined by each gene in the initial prediction set is extended in both directions on the genomic sequence until the neighboring predicted genes are reached. Each such extended region is a gene locus. This procedure often produces thousands or tens of thousands of gene loci on the target species, depending on the size of the genome and the Evigan initial prediction set.

For each proposed gene locus, Evigan was used to generate the *K *best candidate gene models for the gene with the posterior probability for each, by integrating the evidence overlapping with the region. *K *is a parameter passed to the *K *best decoder in Evigan as the maximum number of alternative paths to be generated. If the aggregated evidence at this locus supports less than *K *candidate gene models, all possible models will be generated. The *K*-best decoder [39] in Evigan uses a variation of the Viterbi decoding algorithm [40,41] to search for high probability paths, with *O*(*K N *log *N*) computational complexity where *N *is the size of the standard Viterbi trellis, which is quite efficient. In the original Viterbi decoding implementation of Evigan, an optimal path may contain multiple genes, whereas in the implementation of the *K*-best decoder only single-gene paths are returned. Note that the best candidate in the *K*-best list for a locus may or may not be exactly the same as the initially predicted gene used to identify the locus. In practice, however, discrepancy is rarely observed.

### Ortholog identification

Ortholog pairs between the target species and a reference species are identified by BLASTP [42] reciprocal best hits between the best candidate models (translated into protein products) on the target species and the proteins on the reference species. Specifically, if a gene's best candidate model on the target species and a protein from the reference species are reciprocal best hits by running BLASTP(default parameters, e-value cutoff set as 1e-5), they are considered as an ortholog pair. This is a rather simplified approach for identifying orthologs but in practice it produces reasonably good results. More comprehensive approaches would be searching all candidate models of a gene against the reference proteins or examining multiple species and phylogentic relationships between the species [36,43].

### Reranking features

A variety of features were extracted from candidate gene models, including the posterier probabilities defined by Evigan and various similarity features determined by comparison with orthologous proteins/gene models. Note that these features could readily be expanded to include additional informative similarity features. In the current implementation, six features on a candidate gene model were extracted, as described below. Let *t *and *r *denote a candidate gene model (or its translated protein) on the target species and a protein/gene on the reference species, respectively.

### Posterior probability

Let *p*(*t*) denote *t*'s Evigan posterior probability given the evidence. The probability feature *f*_1_(*t*) is the logarithm of *p*(*t*):

*f*_1_(*t*) = log *p*(*t*)

### Length similarity

Let *l*(*t*) and *l*(*r*) denote the coding sequence length of *t *and *r*. The length similarity feature *f*_2_(*t*) is given by

$$f_{2}(t)=-\log\left(\frac{|l(t)-l(r)|+1}{l(r)+1}\right)$$

The absolute difference in the coding length of the two genes |*l*(*t*) - *l*(*r*)| is normalized by the coding length *l*(*r*) of the reference gene. (Normalizing by the coding length of the target gene model is not a good idea, because it may bias towards target candidate gene models that are very long or short.) The +1 term in the numerator and denominator smoothes the counts.

### Splice count similarity

As with coding length, we also compare the number of splice sites in source and target. Let *s*(*t*) and *s*(*t*) denote the number of splice sites of *t *and *r*. The splice site feature *f*_3_(*t*) is given by

$$f_{3}(t)=-\log\left(\frac{|s(t)-s(r)|+1}{s(r)+1}\right)$$

Again, the +1 term in the numerator and denominator smoothes the counts, and also prevents division by zero.

### Sequence similarity

The sequence similarity feature between *t *and *r *is computed from the alignment score given by DiAlign [44], a multiple sequence alignment program. When two sequences are aligned, DiAlign first searches for multiple gapless local alignments, referred to as segments, and then constructs a global alignment between the two sequences by searching for the best set of consistent segments. In addition to producing gapless local alignments, DiAlign also provides for each segment an alignment score, which is basically the negative logarithm of the probability that two random sequences can be aligned as well as these two sequences. Suppose the coding sequences of *t *and *r *are aligned by DiAlign (translated alignment) and let *A*(*t*, *r*) denote the sum of the alignment scores for the segments constituting the global alignment and *A*(*t*, *r*) is roughly linear to the length of *t *and *r*. The sequence similarity feature *f*_4_(*t*) is given by normalizing the alignment score by the length of *r*, or

$$f_{4}(t)=\frac{A(t,r)}{l(r)}$$

### Shared splice sites

**Figure 3:**
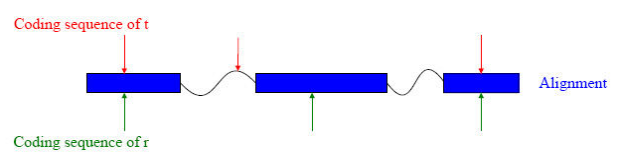
**Infering shared splice sites from alignement**. Blue boxes represent segments (local alignments) produced by DiAlign [44] between coding sequences of two gene models and the wavy lines represent unaligned regions. Arrows represent mapped splice sites. The first and third pairs of overlapping splice sites are identified as shared splice sites.

The segments produced by DiAlign can be used to extract another useful similarity feature: shared splice sites. Figure 3 shows the alignment betweeen the coding sequences of *t *and *r *output by DiAlign, where blue boxes represent gapless local alignments and wavy lines represent unaligned regions. Splice sites of *t *and *r *are mapped to the segments, as shown by the arrows in the figure. If a splice site of *t *and a splice site of *r *are mapped to the same relative position within a segment, as exemplified by the first and third pairs of splice sites in the figure, they are identified as a shared splice site. Let *C*(*t*, *r*) denote the number of shared splice sites identified by the above approach. The shared splice feature *f*_5_(*t*) is given by

$$f_{5}(t)=\log\left(\frac{C(t,r)+1}{s(r)+1}\right)$$

### Signal peptides

A signal peptide feature, *f*_6_(*t*), represents the co-occurence of predicted signal peptide on *t *and *r*. The presence or absence of signal peptides on *t *and *r *is predicted by signalP-3.0 [45]. Let *S*(*t*) and *S*(*r*) denote the presence or absence of signal peptides on *t *and *r*. Then the feature *f*_6_(*t*) is given by

$$f_{6}(t)=\{\begin{array}{ll} 1 & \text{if }S(t)=S(r)=1\\ 0 & \text{otherwise} \end{array}$$

If the reference gene contains a signal peptide, target candidate gene models with signal peptides are preferred; If the reference gene does not contain signal peptide, no preference is imposed on target candidate gene models. The one-sided nature of the feature is motivated by the relatively low abundance of signal peptides and the observation that signal peptide detection algorithms tend to focus on sensitivity rather than specificity. If the reference gene does not have a signal peptide while a target candidate model does, the candidate will not be penalized.

### Scoring function

The features just described are used to compute a score *S*(*t*) for each candidate gene model *t*. The features of *t *are arranged into a feature vector **f**(*t*), and the score is defined by the inner product *S*(*t*) = **f**(*t*)·**w**, where **w **is a weight vector that will be learned from training data. Given *K *candidate gene models *t*_1_, ..., *t*_*K*_, the index of the highest scoring model is given by the decision rule

$$k^{*}=\arg\underset{k=1...K}{\max}S(t_{k})$$

### Weight estimation

The parameter weight vector **w **in the scoring function is estimated from a training set *D *to optimize reranking accuracy using the MIRA online large-margin learning algorithm [46].

The training set *D *= {*e*_1_, ..., *e*_*N*_} is a set of training examples, where each example *e *∈ *D *contains the set of candidate models for a training gene. More specifically, each *e *∈ *D *has the form *e *= {(*t*_*k*_, *q*_*k*_)|*k *= 1, ..., *K*} where *t*_*k *_is a candidate model and *q*_*k *_is the quality of *t*_*k *_relative to the reference annotation. In our experiments, *q*_*k *_is the exon-level F-score (harmonic mean of sensitivity and specificity) for *t*_*k *_relative to the reference annotation genes at *t*_*k*_'s locus.

The MIRA learning algorithm [46] learns **w **by looping over the training examples and updating **w **at each example so that lowest-error candidate model is selected for the example by the decision rule given above. The weight vector **w **is initially the zero vector. The pseudocode "Outline of MIRA update" shows a single cycle of updating the weight vector. At each round, the algorithm fetches an example *e *from the training set, reranks its candidate models and selects the best predicted candidate *t*_k* _using the current weight vector. The true best candidate is denote by $t_{\hat{k}}$, given by the maximum quality assessment. The algorithm updates the weight vector by solving an optimization problem. The goals of the optimization problem are two-fold: keep the new weight vector as close to the current weight vector as possible; and score the true best candidate higher than the predicted candidate by their quality difference $q_{\hat{k}}-q_{k^{*}}$. *C *is a weight factor balancing the two goals, which is set to 5 in the experiments. The algorithm will loop over the examples in the training set until the weight vector does not change significantly.

Outline of MIRA update

Given an example *e *= {(*t*_*k*_, *q*_*k*_)|*k *= 1, ..., *K*} and a current weight vector **w**_*n*_, the updated weight vector **w**_*n*+1 _← MIRA-update(*e*, **w**_*n*_) is computed as follows:

- Use the current weight vector **w**_*n *_to rank the candidate models and select the index for best predicted candidate by k* = arg max_*k *= 1...*K *_**f**(*t*_*k*_)·**w**_*n*_

- Let $\hat{k}$ be the index of the true best candidate $\hat{k}$ = arg max_*k *= 1...*K *_*q*_*k*_

- Find the solution **w**, *ξ *for the following optimization problem:

$$\begin{array}{l} \underset{w,\xi }{\min}||w-w_{n}|{|}^{2}+C\xi \\ \text{subject to }w\cdot f(t_{\hat{k}})\geq w\cdot f(t_{k^{*}})+(q_{\hat{k}}-q_{k^{*}})-\xi ,\xi \geq 0 \end{array}$$

- Set **w**_*n*+1 _= **w**.

It is common practice to consider the average of the updated weight vector at each round as the final output weight vector, because the average weight vector often gives better performance than individual weight vectors [46]. The pseudo-code titled "MIRA algorithm wrapper" shows an algorithm wrapper that calls the MIRA update as a subroutine at each round and outputs a final weight vector.

MIRA algorithm wrapper

Given a training set *D*, the algorithm wrapper computes a weight vector **w **← MIRA-wrapper(D) as follows:

- Initialize the weight vector **w**_0 _← **0**

- Perform the following *N *times:

- Get an example *e *from the training set *D*

- Update the weight vector **w**_*n*+1 _← MIRA-update(*e*, **w**_*n*_)

- Output the average weight vector $w\leftarrow \frac{\sum_{n=1}^{N}w_{n}}{N}$

### Evaluation

For each locus on the target species, Evigan's prediction is always the top gene model from the original candidate list generated by Evigan; ReRanker's prediction is the candidate model with the highest reranking score as described above. Performance of prediction sets is assessed by sensitivity and specificity on exon, transcript and gene level using the Eval program [37] (only coding parts were evaluated). Sensitivity is defined as the fraction of annotated exons (or genes) predicted correctly. Specificity is the fraction of the predicted exons (or genes) that correspond precisely to any exon (or gene) in the curated annotation set. F-score is the harmonic mean of sensitivity and specificity. An exon is considered correct if its boundaries and reading frame are both correct. A gene is counted correct if all of its exons are precisely predicted. For genes with multiple transcripts, sensitivity and specificity were determined at the exon, transcript and gene levels. A transcript is considered correct if all its exons are accurately predicted. A gene is counted correct if one of its transcripts is predicted correctly.

## Authors' contributions

QL, FP and DR designed the experiments. QL performed the experiments and analyzed the results. QL, KC and FP contributed ideas to the algorithms. QL, FP and DR wrote the paper.
